# Correlation between Body Composition and Walking Capacity in Severe Obesity

**DOI:** 10.1371/journal.pone.0130268

**Published:** 2015-06-22

**Authors:** G Correia de Faria Santarém, R de Cleva, Marco Aurélio Santo, Aline Biaseto Bernhard, Alexandre Vieira Gadducci, Julia Maria D’Andrea Greve, Paulo Roberto Santos Silva

**Affiliations:** 1 Department of Gastroenterology, University of São Paulo Medical School, São Paulo, São Paulo, Brazil; 2 Department of Orthopedics and Traumatology, University of São Paulo Medical School, São Paulo, São Paulo, Brazil; Children's National Medical Center, Washington, UNITED STATES

## Abstract

**Background:**

Obesity is associated with mobility reduction due to mechanical factors and excessive body fat. The six-minute walk test (6MWT) has been used to assess functional capacity in severe obesity.

**Objective:**

To determine the association of BMI, total and segmental body composition with distance walked (6MWD) during the six-minute walk test (6MWT) according to gender and obesity grade.

**Setting:**

University of São Paulo Medical School, Brazil; Public Practice.

**Methods:**

Functional capacity was assessed by 6MWD and body composition (%) by bioelectrical impedance analysis in 90 patients.

**Results:**

The mean 6MWD was 514.9 ± 50.3 m for both genders. The male group (M: 545.2 ± 46.9 m) showed a 6MWD higher (p = 0.002) than the female group (F: 505.6 ± 47.9 m). The morbid obese group (MO: 524.7 ± 44.0 m) also showed a 6MWD higher (p = 0.014) than the super obese group (SO: 494.2 ± 57.0 m). There was a positive relationship between 6MWD and fat free mass (FFM), FFM of upper limps (FFM_UL), trunk (FFM_TR) and lower limbs (FFM_LL). Female group presented a positive relationship between 6MWD and FFM, FFM_UL and FFM_LL and male group presented a positive relationship between 6MWD and FFM_TR. In morbid obese group there was a positive relationship between 6MWD with FFM, FFM_UL, FFM_TR and FFM_LL. The super obese group presented a positive relationship between 6MWD with FFM, FFM_TR and FFM_LL.

**Conclusions:**

Total and segmental FFM is associated with a better walking capacity than BMI.

## Introduction

The obesity has doubled worldwide and is considered the fifth risk factor for mortality. Future projections estimates a 33% increase in obesity and 130% increase in severe obesity prevalence in 2030 [1]. Obesity is associated with a reduction in individual mobility, aggravating previous sedentary lifestyle [2,3]. Activities of daily living (ADL) are impaired due not only to excessive body fat accumulation but also to mechanical factors that might reduce walking capacity. The ability to walk is a simple measure of physical function and an important component of quality of life, since it reflects the capacity to perform day-to-day activities [4,5]. The distance traveled during the six-minute walk test (6MWD) on a horizontal surface is an easy, validated and inexpensive method to evaluate individual physical function [6,7].

Previous studies [8,9] demonstrated a negative relationship between the body mass index (BMI) and 6MWD. Although BMI is an index widely accepted to classify the severity of obesity, it is not considered the best index to determine body composition or fat free mass [10,11].

Some researchers suggested that women have greater total body fat than men for the same BMI [12]. Obese females tend to accumulate adipose tissue in lower extremity (gynoid adiposity) [13], while males tend to accumulate adipose tissue in the abdominal area (android adiposity). This segmental fat distribution can affect physical function [14].

Obese and super obese (BMI between 50 and 60 kg/m^2^) patients adapt to their greater body mass by slowing down walking velocity [2,5,15]. The severely obese tend to oscillate their trunk when walking and to increase the distance between ankles when stopping to compensate the extra body mass [15].

To the best of our knowledge there are no studies correlating the 6MWD with segmental body composition in severe obesity. The aim of our study was to correlate the 6MWD with BMI, total and segmental body composition in severe obesity according to gender and obesity grade.

## Materials and Methods

### Participant recruitment

Eligibility criteria for participants to be admitted to this study were: BMI between 40 and 60 kg/m^2^, aged between 18 and 60 years, test Timed Up and Go (TUG) ≤ 10 seconds [16] and ability to understand and perform all procedures proposed. The study protocol was performed according to the ethical recommendations of the Declaration of Helsinki and was approved by the Ethical Committee of the Hospital das Clínicas, University of São Paulo Medical School (protocol number 01038912.6.0000.0068) and conducted after the participants signed the consent form.

Subjects were selected according to the exclusion criteria defined as follows: BMI > 60 kg/m^2^ (n = 3), cognitive disorders (n = 1), neuromuscular, musculoskeletal or rheumatologic diseases, use of artificial devices (orthesis), presence of lung disease (spirometry test that did not reach 80% of predicted values) (n = 2) [17], TUG test > 10 s (n = 3), systemic corticosteroid use, absolute contraindication for the 6MWT according to the guidelines of the American Thoracic Society (ATS) [6] and subjects who did not agree to participate in the study (n = 7). On the basis of these criteria, 16 participants out of 106 were excluded. Then, 90 participants (69 women, age: 40.5 ± 9.6 years, BMI: 47.6 ± 5.4 kg/m^2^, and 21 men, age: 38.8 ± 10.7 years, BMI: 48.2 ± 4.9 kg/m^2^) were included and classified according to BMI in morbid obese (MO: BMI between 40 and 49.9 kg/m^2^) and super obese (SO: BMI between 50 and 60 kg/m^2^) groups.

### Spirometry Test

Data were collected for identification, anthropometric measures and lung function test. Spirometry was assessed by maximal respiratory maneuvers and flow-volume curves (MIR, Spirobank II, Roma, Italy). Participants breathed in a sitting position through a disposable mouthpiece positioned between the teeth and lips, ensuring that no leaks occurred during forced expiration. The adopted technical procedures and criteria for acceptability and reproducibility followed the recommendations of the European Respiratory Society/American Thoracic Society (ERS/ATS) [17]. The maneuver of forced vital capacity was performed 3 times and the best performance curve was selected. Forced vital capacity (FVC), forced expiratory volume in one second (FEV_1_), FEV_1_/FVC ratio, and mean forced expiratory flow between 25 and 75% of the volume of FVC (FEF_25-75%_) were assessed in absolute and predicted values [18].

### Anthropometric measurements and body mass composition

Body composition was evaluated by anthropometry and bioelectrical impedance analysis (BIA). Height and body weight were measured with participants to the nearest 0.5 cm and 0.1 kg, respectively. Body mass index (BMI) was calculated using the formula: [weight (kg)/height (m^2^)]. Body composition was determined by BIA (Biospace Co., InBody 230, USA) under constant conditions (proper hydration and same time of day). The participant was positioned in orthostatic position on a platform with lower electrodes for feet and the hands holding the upper electrodes. This equipment can measure the impedance of each body segment by using 2 different frequencies (20 KHz and 100 KHz). The chemical composition of lean body mass is conventionally assumed to be constant, with a density of 1.1 kg/m^3^ at a temperature of 37°C and body water content of 73.3%. Thus, the fat free mass of the upper trunk and lower limbs were calculated by multiplying the value of water volume in the upper extremities (the sum of the right and left), trunk and lower limbs (the sum of the right and left) by 1.37. Each segmental of fat free mass was divided by the total body weight for body fat free mass estimate for the segment body weight. BIA determined in percentage values (%): fat mass (FM), fat free mass (FFM), fat free mass of upper limps (FFM_UL), fat free mass of trunk (FFM_TR), fat free mass of lower limbs (FFM_LL), fat mass of upper limps (FM_UL), fat mass of trunk (FM_TR) and fat mass of lower limbs (FM_LL).

### Six-minute walk test

The 6MWT was conducted on a plane surface in an undisturbed 36 meters (m) long corridor marked every 3 m with colored tape on the floor. The test was conducted according to the recommendations of ATS [6]. Participants were instructed to walk the longest distance possible, were allowed to stop and rest during the test, but were instructed to resume walking as soon as they felt capable. Standardized encouragement (for example: “You are doing well. You have 5 minutes to go.”) and announcement of remaining time were given to all participants [6]. The basal heart rate (HR), oxy-hemoglobin saturation (Ana Wiz, ANP 100, China), fatigue and dyspnea scores (Borg scale) [19] and arterial blood pressure (Omron Healthcare Co, LTD, Japan) were obtained from all participants before and after the test. The test was interrupted only for the following reasons: diaphoresis, pale or ashen appearance, leg cramps, staggering, chest pain and intolerance dyspnea.

### Statistical analysis

Sample size was estimated using a linear regression analysis between 6MWD (response variable) with gender and obesity grade. The minimum sample size was calculated to be at least 90 subjects. A p < 0.05 value was considered as significant.

All data were presented as mean, standard deviation and 95% confidence intervals.

The association of the studied variables (6MWD with variables: BMI, FFM, FM, FFM_UL, FFM_TR, FFM_LL, FM_UL, FM_TR, FM_LL) was evaluated through the Pearson and Spearman correlation. Unpaired T-test and Mann-Whitney was used to determine intergroup (F and M, MO and SO groups) differences in numerical data.

## Results

### Spirometry Test

Ninety severe obese adults (age: 40.1 ± 9.8 years, BMI: 47.7 ± 5.2 kg/m^2^) were recruited at Metabolic and Bariatric Surgery Unit, Hospital das Clínicas, University of São Paulo Medical School.

All participants included in this study did not show any significant alterations in spirometric values as FVC, FEV_1_, FEV_1_/FVC and FEF_25-75%_. The spirometric data are summarized in Table 1.

**Table 1:** Spirometric data (absolute and predicted values) of the participants.

Variables	Total (n = 90)
FVC (L)	3.2 ± 0.9
FVC (%)	97.5 ± 12.9
FEV_1_ (ml)	2501.0 ± 875.6
FEV_1_ (%)	94.3 ± 11.7
FEV_1_/FVC	81.2 ± 7.5
FEV_1_/FVC (%)	100.1 ± 8.9
FEF_25-75%_ (L/s)	2.9 ± 1.0
FEF_25-75%_ (%)	88.5 ± 7.4

Results are expressed as mean ± SD.

FVC, forced vital capacity; FVC, percentage of predicted FVC; FEV_1_, forced expiratory volume in one second; FEV_1_, percentage of predicted VEF_1_; FEV_1_/FVC, forced expiratory volume in one second/ forced vital capacity; FEV_1_/FVC, percentage of predicted forced expiratory volume in one second/forced vital capacity; FEF_25-75%_, forced expiratory flow between 25 and 75% FVC; FEF_25-75%_, percentage of predicted forced expiratory flow between 25 and 75% FVC.

### Anthropometric measurements and body mass composition

The anthropometry data of the study participants are presented in Table 2. There was no significant difference (p = 0.557) in BMI between the male (M) and female (F) groups (M: 48.2 ± 4.9, F: 47.6 ± 5.4). There was a significant difference (p < 0.001) between the M and F groups in terms of FFM, FM, FFM_UL, FFM_TR, FFM_LL, FM_UL, FM_TR and FM_LL.

**Table 2:** Anthropometric characteristics and body composition of the study participants determined by BIA.

Variables	Total	F	M	MO	SO
	(n = 90)	(n = 69)	(n = 21)	(n = 61)	(n = 29)
Height (cm)	162.9 ± 9.3	159.6 ± 6.3	173.6 ± 9.3**	162.8 ± 8.3	163.1 ± 11.1
FFM (%)	49.3 ± 4.1	48.0 ± 3.0	53.5 ± 4.6**	50.5 ± 4.1**	46.8 ± 2.8
FFM_UL (%)	32.2 ± 6.4	31.1 ± 5.3	35.8 ± 8.2**	35.1 ± 5.2**	26.2 ± 4.1
FFM_TR (%)	51.0 ± 3.4	49.8 ± 2.6	54.9 ± 2.7**	51.2 ± 3.3	50.7 ± 3.6
FFM_LL (%)	50.8 ± 5.2	49.1 ± 3.9	56.5 ± 5.1**	52.4 ± 4.7**	47.4 ± 4.8
BMI (kg/m^2^)	47.7 ± 5.2	47.6 ± 5.4	48.2 ± 4.9	44.7 ± 2.9	54.2 ± 2.4**
FM (%)	50.7 ± 4.1	52.0 ± 3.0**	46.5 ± 4.6	49.6 ± 4.1	53.3 ± 2.8**
FM_UL (%)	67.8 ± 6.4	68.9 ± 5.3**	64.2 ± 8.2	64.9 ± 5.2	73.9 ± 4.1**
FM_TR (%)	49.0 ± 3.8	50.2 ± 2.6**	45.1 ± 2.7	48.8 ± 3.3	49.3 ± 3.6
FM_LL (%)	49.2 ± 5.3	51.0 ± 3.9**	43.5 ± 5.1	47.6 ± 4.7	52.6 ± 4.8**

Results are expressed as mean ± SD.

F, female group; M, male group; MO, morbid obese group; SO, super obese group; FFM, fat free mass; FFM_UL, fat free mass of upper limbs; FFM_TR, fat free mass of trunk; FFM_LL, fat free mass of lower limbs; BMI, body mass index; FM, fat mass; FM_UL, fat mass of upper limbs; FM_TR, fat mass of trunk; FM_LL, fat mass of lower limbs;

**p < 0.001 between gender (M and F groups) and obesity grade (MO and SO groups).

Of the 90 participants studied, 61 (67.8%) were MO (BMI: 44.7 ± 2.9 kg/m^2^) and 29 (32.2%) were SO (BMI: 54.2 ± 2.4 kg/m^2^). There was a significant difference (p < 0.001) between the SO and MO groups in terms of FFM, FFM_UL, FFM_LL, FM, FM_UL and FM_LL. The FFM_TR and FM_TR were similar in both groups (p = 0.506).

### Six-minute walk test

The 6MWT results are summarized in Table 3. All participants completed the 6MWT without premature end or breaks, and no complications have occurred during the test. The total 6MWD was 514.9 ± 50.3 m. The M group (545.2 ± 46.9 m) showed a 6MWD significantly higher (p = 0.002) than the F group (505.6 ± 47.9 m). The MO group (524.7 ± 44.0 m) also showed a 6MWD significantly higher (p = 0.014) than the SO group (494.2 ± 57.0 m).

**Table 3:** 6MWT parameters of the study participants.

Variables	Total	F	M	MO	SO
	(n = 90)	(n = 69)	(n = 21)	(n = 61)	(n = 29)
6MWD (m)	514.9 ± 50.3	505.6 ± 47.9	545.2 ± 46.9*	524.7 ± 44.0*	494.2 ± 57.0
HR (rpm) baseline	83.4 ± 13.4	83.1 ± 12.6	84.8 ± 16.4	83.0 ± 12.7	84.1 ± 15.3
After 6 MWT	147.7±19.0**	147.5 ± 17.4**	148.2 ± 24.5**	147.4 ± 18.9**	147.9 ± 19.4**
SatO2 (%) baseline	96.3 ± 1.9	96.5 ± 1.8	95.6 ± 2.2	96.5 ± 1.6	95.9 ± 2.5
After 6 MWT	96.7 ± 1.6	96.9 ± 1.5	96.0 ± 1.6	96.7 ± 1.4	96.7 ± 2.0
SBP (mmHg) baseline	133.9 ± 18.1	132.2 ± 17.4	140.1 ± 20.0	134.0 ± 20.2	133.2 ± 13.8
After 6 MWT	148.7 ± 20.5**	146.5 ± 20.0**	157.1 ± 20.5**	149.7 ± 19.8**	149.4 ± 21.3**
DBP (mmHg) baseline	84.6 ± 15.0	84.3 ± 15.1	85.6 ± 14.9	83.9 ± 11.2	82.9 ± 2.5
After 6 MWT	84.6 ± 11.0	84.2 ± 10.4	85.7 ± 13.3	85.4 ± 11.7	83.2 ± 2.3
Dyspnea score baseline	1.5 ± 2.1	1.7 ± 2.2	0.9 ± 1.6	1.8 ± 2.7	2.0 ± 2.0
After 6 MWT	4.2 ± 2.8**	4.7 ± 2.7**	2.47 ± 2.1**	4.1 ± 2.9**	4.6 ± 2.4**
Fatigue score baseline	2.1 ± 2.8	2.4 ± 3.1	1.2 ± 1.6	2.2 ± 3.2	2.3 ± 2.4
After 6 MWT	4.2 ± 3.1**	4.6 ± 3.1**	2.5 ± 2.2**	3.9 ± 3.0**	4.7 ± 3.2**

Results are expressed as mean ± SD.

6MWD, six-minute walk distance; HR, heart rate; SatO_2_, oxy-hemoglobin saturation; SBP, systolic blood pressure; DBP, diastolic blood pressure;

*p < 0.001 between gender (M and F groups) and obesity grade (MO and SO groups);

**p < 0.001 between baseline and after 6MWT.

### Correlation between body composition and 6MWD

The correlations between body composition and 6MWD are summarized in Table 4. A positive correlation was found between 6MWD and FFM, FFM_UL, FFM_TR, FFM_LL and a significant negative correlation were found between BMI, FM, FM_UL, FM_TR and FM_LL.

**Table 4:** Correlation between 6MWD and body mass composition.

Variables	Total (n = 90)		F (n = 69)		M (n = 21)		MO (n = 61)		SO (n = 29)	
	r	95% CI	r	95% CI	r	95% CI	r	95% CI	r	95% CI
FFM (%)	0.5***	0.3; 0.7	0.4*	0.1; 0.6	0.4	-0.1; 0.7	0.4*	0.2; 0.7	0.5*	0.1; 0.7
FFM_UL (%)	0.4***	0.2; 0.6	0.4**	0.1; 0.6	0.2	-0.3; 0.7	0.4**	0.1; 0.6	0.1	-0.3; 0.5
FFM_TR (%)	0.3*	0.1; 0.5	0.0	-0.3; 0.3	0.6*	0.2; 0.9	0.3*	0.0; 0.6	0.4*	0.0; 0.7
FFM_LL (%)	0.5***	0.3; 0.7	0.4*	0.1; 0.6	0.4	-0.1; 0.7	0.4**	-0.2; -0.7	0.4*	0.0; 0.7
BMI (kg/m^2^)	-0.3**	-0.6; -0.2	-0.5***	-0.7; -0.3	-0.1	-0.6; 0.4	-0.2	-0.6; 0.0	-0.2	-0.6; 0.2
FM (%)	-0.5***	-0.7; -0.3	-0.4*	-0.6; -0.1	-0.4	-0.7; 0.1	-0.4*	-0.7; -0.2	-0.5*	-0.7; -0.1
FM_UL (%)	-0.4***	-0.6; -0.2	-0.4**	-0.6; -0.1	-0.2	-0.7; 0.3	-0.4**	-0.6; -0.1	-0.1	-0.5; 0.3
FM_TR (%)	-0.3*	-0.6; -0.2	0.0	-0.3; 0.3	-0.6*	-0.9; -0.2	-0.3*	-0.6; 0.0	-0.4*	-0.7; 0.0
FM_LL (%)	-0.5***	-0.7; -0.3	-0.4*	-0.6; -0.1	-0.4	-0.7; 0.1	-0.4**	-0.7; -0.2	-0.4*	-0.7; 0.0

BMI, body mass index; FFM, fat free mass; FM, fat mass; FFM_UL, fat free mass of upper limbs; FFM_TR, fat free mass of trunk; FFM_LL, fat free mass of lower limbs; FM_UL, fat mass of upper limbs; FM_TR, fat mass of trunk; FM_LL, fat mass of lower limbs; 95% CI, 95% confidence intervals;

*p < 0.05;

**p < 0.001;

***p < 0.000.1.

In female group the 6MWD presented a significant positive correlation with FFM, FFM_UL, FFM_LL and a significant negative correlation with BMI, FM, FM_UL and FM_LL. In male group, 6MWD was significantly positive correlated with FFM_TR and was significantly negative correlated with FM_TR.

In morbid obese group, a significant positive correlation was found between 6MWD and FFM, FFM_UL, FFM_TR, FFM_LL and a significant negative correlation was found between FM, FM_UL, FM_TR, FM_LL.

In super obese group the 6MWD presented a significant positive correlation with FFM, FFM_TR and FFM_LL and a significant negative correlation with FM, FM_TR and FM_LL.

## Discussion

The objective of our study was to correlate the performance in 6MWT with BMI, total and segmental body composition according to gender and obesity grade. Severe obesity affects walking ability and the functional capacity of this population. The importance of functional assessment of morbidly obese patients is well documented [4,20,21]. Nowadays, the 6MWT is being used to evaluate the functional capacity of morbid obese patients to realize their ADL [8,9,21,22].

In the present study, the mean 6MWD (514.9 ± 50.3 m) was significantly greater than previously studies in morbid obese populations [9,20,22]. Our severe obese patients presented a functional performance similar to healthy individuals [7,23,24]. In previous studies [9,25], morbid obese individuals presented functional capacity similar to elderly population [26] or to patients with cardiac [27] and pulmonary diseases [28]. Only two studies with morbid obese [29,30] patients present similar performance. Some hypotheses may explain the better functional performance observed in our study. Our participants could present a better functional capacity, but it seems unlikely since our group presented characteristics (BMI, gender, age) representative of MO patients included in other studies. Another hypothesis could be methodological problems during the 6MWT in other studies such as small corridor (less than 30 m), inappropriate verbal stimulus or incorrect measurement of distance traveled, inadvertent inclusion of subjects with cardiopulmonary diseases undiagnosed or inclusion of participants with physical limitations that could reduce 6MWD such as neuromuscular, musculoskeletal or rheumatologic disorders.

We demonstrate a significant negative relationship between the 6MWD and BMI only in women [8,25]. In other previous studies these correlation was less or more marked [26,22] than our results. Nevertheless, BMI was not a good predictor of functional capacity in men and when obesity was graded. 6MWD presented a positive correlation with FFM. These evidences confirm previous findings in subjects with different ages and degrees of obesity [31]. Our study also demonstrated that the FFM might better explain the 6MWD than BMI.

We also observed significant differences in mean 6MWD of F and M, MO and SO groups [8,25,30]. Our data showed a better functional capacity in men than women, which may be explained by a difference in body fat distribution and height [26,22]. In our study, the fat mass of female group predominates in the hip and thighs (gynoid obesity), making the ability to walk less functional than in male group (android obesity). In contrast, FFM of male group was significantly greater than female and concentrated in trunk and lower limbs. Another important issue is the fact that men were taller than women, and a taller height is associated with a longer stride walking. 6MWD showed a positive association with FFM_TR in men and with FFM_UL and FFM_LL in women. Studies have suggested that women with high body fat in lower limbs present biomechanical disadvantage in comparison with men (central obesity). Adipose deposit in legs alters weight bearing at the knee and consequently, reduces walk capacity with a negative impact in functional capacity [14]. A high adipose tissue accumulation in the abdominal area changes the center of gravity position, determining an alteration in balance control.

The mass distribution may also explain the significant differences in mean 6MWD of MO and SO groups. In MO group, the 6MWD showed a positive association with FFM, FFM_UL, FFM_TR, FFM_LL and with FFM, FFM_TR and FFM_LL in SO group.

Our data suggest that functional capacity assessed by the 6MWT is not only related to the segmental body composition but also to adequate postural control, balance and coordinated movement.

## Conclusions

Total and segmental FFM are associated with a better walking capacity than BMI. The FFM of upper and lower limbs in women, FFM of trunk in men, FFM of upper and lower limbs in the morbid obese and FFM of trunk and lower limbs are associated with best functional performance in severe obesity. Physical exercises specifically planned for severe obese patients may prevent loss of fat free mass.

## Supporting Information

S1 Table Spirometric data (absolute and predicted values) of the participants. Results are expressed as mean ± SD. FVC. forced vital capacity; FVC, percentage of predicted FVC; FEV_1_, forced expiratory volume in one second; FEV_1_, percentage of predicted VEF_1_; FEV_1_/FVC, forced expiratory volume in one second/ forced vital capacity; FEV_1_/FVC, percentage of predicted forced expiratory volume in one second/forced vital capacity; FEF_25-75%_, forced expiratory flow between 25 and 75% FVC; FEF_25-75%_, percentage of predicted forced expiratory flow between 25 and 75% FVC.
(DOC)

S2 Table Anthropometric characteristics and body composition of the study participants determined by BIA. Results are expressed as mean ± SD. F, female group; M, male group; MO, morbid obese group; SO, super obese group; FFM, fat free mass; FFM_UL, fat free mass of upper limbs; FFM_TR, fat free mass of trunk; FFM_LL, fat free mass of lower limbs; BMI, body mass index; FM, fat mass; FM_UL, fat mass of upper limbs; FM_TR, fat mass of trunk; FM_LL, fat mass of lower limbs; **p < 0.001 between gender (M and F groups) and obesity grade (MO and SO groups).
(DOC)

S3 Table 6MWT parameters of the study participants. Results are expressed as mean ± SD. 6MWD, six-minute walk distance; HR, heart rate; SatO_2_, oxy-hemoglobin saturation; SBP, systolic blood pressure; DBP, diastolic blood pressure; *p < 0.001 between gender (M and F groups) and obesity grade (MO and SO groups); **p < 0.001 between baseline and after 6MWT.
(DOC)

S4 Table Correlation between 6MWD and body mass composition. BMI, body mass index; FFM, fat free mass; FM, fat mass; FFM_UL, fat free mass of upper limbs; FFM_TR, fat free mass of trunk; FFM_LL, fat free mass of lower limbs; FM_UL, fat mass of upper limbs; FM_TR, fat mass of trunk; FM_LL, fat mass of lower limbs; 95% CI, 95% confidence intervals; *p < 0.05; **p < 0.001; ***p < 0.000.1.
(DOC)
