# Supplementary material for: Correlation between Body Composition and Walking Capacity in Severe Obesity
Source: PLoS One. 2015 Jun 22;10(6):e0130268. doi: 10.1371/journal.pone.0130268 (PMC4476574; doi:10.1371/journal.pone.0130268)
Supplement: S1 Table — Results are expressed as mean ± SD. FVC. forced vital capacity; FVC, percentage of predicted FVC; FEV1, forced expiratory volume in one second; FEV1, percentage of predicted VEF1; FEV1/FVC, forced expiratory volume in one second/ forced vital capacity; FEV1/FVC, percentage of predicted forced expiratory volume in one second/forced vital capacity; FEF25-75%, forced expiratory flow between 25 and 75% FVC; FEF25-75%, percentage of predicted forced expiratory flow between 25 and 75% FVC. (DOC) [file pone.0130268.s001.doc]

**S1 Table. Spirometric data (absolute and predicted values) of the participants.** Results are expressed as mean ± SD. FVC. forced vital capacity; FVC, percentage of predicted FVC; FEV_1_, forced expiratory volume in one second; FEV_1_, percentage of predicted VEF_1_; FEV_1_/FVC, forced expiratory volume in one second/ forced vital capacity; FEV_1_/FVC, percentage of predicted forced expiratory volume in one second/forced vital capacity; FEF_25-75%_, forced expiratory flow between 25 and 75 % FVC; FEF_25-75%_, percentage of predicted forced expiratory flow between 25 and 75 % FVC.
