# Supplementary material for: Correlation between Body Composition and Walking Capacity in Severe Obesity
Source: PLoS One. 2015 Jun 22;10(6):e0130268. doi: 10.1371/journal.pone.0130268 (PMC4476574; doi:10.1371/journal.pone.0130268)
Supplement: S2 Table — Results are expressed as mean ± SD. F, female group; M, male group; MO, morbid obese group; SO, super obese group; FFM, fat free mass; FFM_UL, fat free mass of upper limbs; FFM_TR, fat free mass of trunk; FFM_LL, fat free mass of lower limbs; BMI, body mass index; FM, fat mass; FM_UL, fat mass of upper limbs; FM_TR, fat mass of trunk; FM_LL, fat mass of lower limbs; **p < 0.001 between gender (M and F groups) and obesity grade (MO and SO groups). (DOC) [file pone.0130268.s002.doc]

**S2 Table. Anthropometric characteristics and body composition of the study participants determined by BIA.** Results are expressed as mean ± SD. F, female group; M, male group; MO, morbid obese group; SO, super obese group; FFM, fat free mass; FFM_UL, fat free mass of upper limbs; FFM_TR, fat free mass of trunk; FFM_LL, fat free mass of lower limbs; BMI, body mass index; FM, fat mass; FM_UL, fat mass of upper limbs; FM_TR, fat mass of trunk; FM_LL, fat mass of lower limbs; ^**^p < 0.001 between gender (M and F groups) and obesity grade (MO and SO groups).
