# Supplementary material for: Correlation between Body Composition and Walking Capacity in Severe Obesity
Source: PLoS One. 2015 Jun 22;10(6):e0130268. doi: 10.1371/journal.pone.0130268 (PMC4476574; doi:10.1371/journal.pone.0130268)
Supplement: S3 Table — Results are expressed as mean ± SD. 6MWD, six-minute walk distance; HR, heart rate; SatO2, oxy-hemoglobin saturation; SBP, systolic blood pressure; DBP, diastolic blood pressure; *p < 0.001 between gender (M and F groups) and obesity grade (MO and SO groups); **p < 0.001 between baseline and after 6MWT. (DOC) [file pone.0130268.s003.doc]

**S3 Table. 6MWT parameters of the study participants.** Results are expressed as mean ± SD. 6MWD, six-minute walk distance; HR, heart rate; SatO_2_, oxy-hemoglobin saturation; SBP, systolic blood pressure; DBP, diastolic blood pressure; ^*^p < 0.001 between gender (M and F groups) and obesity grade (MO and SO groups); ^**^p < 0.001 between baseline and after 6MWT.
