# Supplementary material for: Correlation between Body Composition and Walking Capacity in Severe Obesity
Source: PLoS One. 2015 Jun 22;10(6):e0130268. doi: 10.1371/journal.pone.0130268 (PMC4476574; doi:10.1371/journal.pone.0130268)
Supplement: S4 Table — BMI, body mass index; FFM, fat free mass; FM, fat mass; FFM_UL, fat free mass of upper limbs; FFM_TR, fat free mass of trunk; FFM_LL, fat free mass of lower limbs; FM_UL, fat mass of upper limbs; FM_TR, fat mass of trunk; FM_LL, fat mass of lower limbs; 95% CI, 95% confidence intervals; *p < 0.05; **p < 0.001; ***p < 0.000.1. (DOC) [file pone.0130268.s004.doc]

**S4 Table. Correlation between 6MWD and body mass composition.** BMI, body mass index; FFM, fat free mass; FM, fat mass; FFM_UL, fat free mass of upper limbs; FFM_TR, fat free mass of trunk; FFM_LL, fat free mass of lower limbs; FM_UL, fat mass of upper limbs; FM_TR, fat mass of trunk; FM_LL, fat mass of lower limbs; 95% CI, 95% confidence intervals; ^*^p < 0.05; ^**^p < 0.001; ^***^p < 0.000.1.
